# *Hippobosca longipennis *- a potential intermediate host of a species of *Acanthocheilonema *in dogs in northern India

**DOI:** 10.1186/1756-3305-4-143

**Published:** 2011-07-22

**Authors:** Puteri Azaziah Megat Abd Rani, Glen T Coleman, Peter J Irwin, Rebecca J Traub

**Affiliations:** 1 School of Veterinary Science, The University of Queensland, Gatton, Queensland, Australia; 2 School of Veterinary and Biomedical Science, Murdoch University, Western Australia, Australia; 3 Faculty of Veterinary Medicine, Universiti Putra Malaysia, Malaysia

## Abstract

**Background:**

*Hippobosca longipennis *(the 'dog louse fly') is a blood sucking ectoparasite found on wild carnivores such as cheetahs and lions and domesticated and feral dogs in Africa, the Middle East and Asia, including China. Known as an intermediate host for *Acanthocheilonema dracunculoides *and a transport host for *Cheyletiella yasguri*, it has also been suggested that *H. longipennis *may be a vector for other pathogens, including *Acanthocheilonema *sp.? nov., which was recently reported to infect up to 48% of dogs in northern India where this species of fly is known to commonly infest dogs. To test this hypothesis, hippoboscid flies feeding on dogs in Ladakh in northern India were collected and subjected to microscopic dissection.

**Results:**

A total of 12 infective larvae were found in 10 out of 65 flies dissected; 9 from the head, 2 from the thorax and 1 from the abdomen. The larvae averaged 2, 900 (± 60) μm in length and 34 (± 5) μm in width and possessed morphological features characteristic of the family Onchocercidae. Genetic analysis and comparison of the 18S, ITS-2, 12S and cox-1 genes confirmed the identity of the larvae as the *Acanthocheilonema *sp.? nov. reported in dogs in Ladakh.

**Conclusion:**

This study provides evidence for a potential intermediate host-parasite relationship between *H. longipennis *and the canine *Acanthocheilonema *sp.? nov. in northern India.

## Background

Hippoboscids are highly specialised larviparous ectoparasitic flies, spending all or most of their adult life within the fur or among feathers of their mammal and avian hosts [1]. The family Hippoboscidae is divided into three subfamilies; Lipopteninae, Ornithomyiinae and Hippoboscinae. This family represents one of the most important blood-sucking insect groups of birds and ruminants [2], however comparatively little attention has been paid to their role as an ectoparasite of dogs and their potential as an intermediate host for canine parasites.

Wild and domestic canids represent the preferred/primary hosts for *Hippobosca longipennis *[3]. The geographical distribution of *H. longipennis *includes arid and semi arid regions of southern Europe, Africa, the Middle East and Asia, including China and India [4-6]. In 1970, *H. longipennis *was introduced into the United States after infested captive cheetahs were imported from east Africa [7] and the flies were subsequently detected on cheetahs at safari parks in Texas, Georgia and Oregon. Eradication efforts were successful and since then there is no evidence that this species has become established in the United State or elsewhere in the New World [6-8]. *Hippobosca longipennis *has been reported to alight on humans [5] and occasionally bite [3], but the extent of their ability to feed on humans is unknown. The fly has been reported infesting Indian dogs since 1966 and has been reported in Uttar Pradesh, Himachal Pradesh, Jammu and Kashmir, Punjab, Assam, West Bengal and the eastern zone of Maharashtra state [3,5,9,10].

With the exception of *Dirofilaria immitis *and *Dirofilaria repens*, other species of canine filarial infections are generally asymptomatic, but microfilariae have been associated with skin hypersensitivity reactions [11,12]. Adult filarial nematodes of the genus *Acanthocheilonema *are usually found in the body cavities and/or subcutaneous tissues of their mammalian host. The public health significance of the genus *Acanthocheilonema *appears to be minimal, with only one case of an adult female *Acanthocheilonema reconditum *reported in the eye of a human, in Australia [13]. Fleas and lice are intermediate hosts for *A. reconditum *[14]. Ticks, lice [15,16] and, in Algeria, *H. longipennis*, have all been proposed as intermediate hosts for *Acanthocheilonema dracunculoides *[17]. The development of larvae in these intermediate hosts occurs mostly in the fat-body cells and infective larvae migrate to the mouthparts to facilitate their transmission [18,19].

Recently we reported the discovery of a canine filarial worm that was referred to erroneously as *Acanthocheilonema ladakhii *(currently *nomen nudum*), in 48% of a dog population sampled in northern India [20]. To date very little is known about the clinical significance of this parasite except that its microfilariae may be confused with those of *D. immitis*, especially by minimally trained personnel [21], and that almost all the dogs in this region were heavily infested with *H. longipennis *[20].

The aim of this study was to report the occurrence of the newly reported *Acanthocheilonema *parasite in *H. longipennis *in dogs in northern India, using a combination of conventional and molecular diagnostic techniques. This species of *Acanthocheilonema *(currently *nomen nudum*) will be referred to as *Acanthocheilonema *sp.? nov. throughout this manuscript.

## Materials and methods

### Study site and sampling

**Figure 1:**
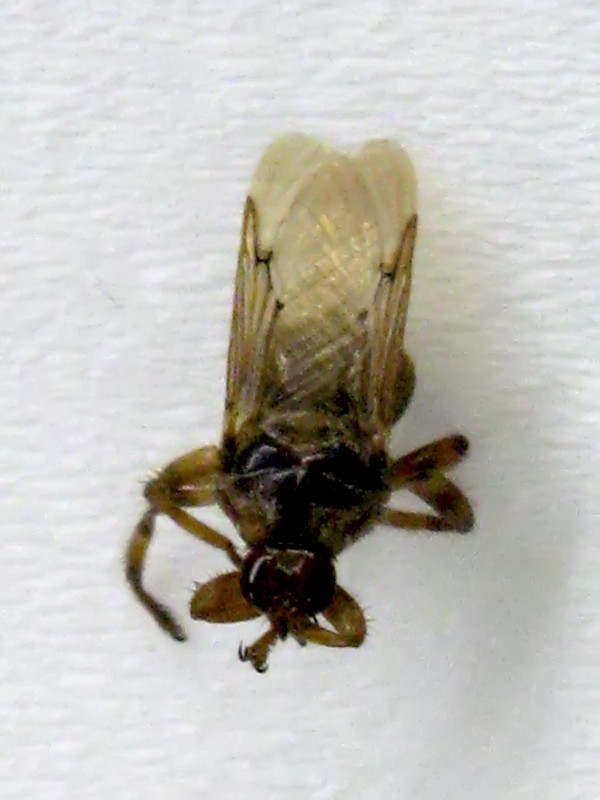
**Adult specimen of the dog fly, *Hippobosca longipennis *found infesting dogs in Ladakh, India**.

Approximately 200 adult flies belonging to the genus *Hippobosca *(Figure 1) were manually removed from dogs presented to the Street Dog Sterilisation Programme in Ladakh, India, from June to September 2008. This collaborative program, involving the Ladakh Animal Care Society and Vets Beyond Borders, aims to stabilise the street dog population and helps to control rabies and other canine zoonoses. The flies were deposited into screw-cap tubes and immediately fixed in 70% ethanol.

### Identification and dissection

Flies were examined using a stereomicroscope and identified morphologically according to criteria specified by Bequaert [3]. The flies were then individually dissected; after discarding legs and wings, each specimen was divided into three body sections (head, thorax and abdomen). Flies were dissected in phosphate-buffered saline (PBS) using curved-bladed Vannas scissors (ProSciTech, Australia).

All isolated nematode larvae were examined microscopically at ×200 and ×400 magnification for morphological identification with reference to an atlas for infective larvae of filarial parasites [22]. The larvae were measured using a BH-2 microscope (Olympus, Japan) with a calibrated eye micrometer and photographed using a DP12 digital microscope camera (Olympus, Japan).

### DNA Extraction, PCR assays and DNA sequencing

DNA extraction was performed on collected larvae using a commercial kit (DNeasy Tissue Kit; Qiagen) and subjected to PCR for genus and species identification. A single DNA sample of canine blood positive for microfilariae of *Acanthocheilonema *sp.? nov. was randomly selected from the authors' previous study [20] and was genetically characterised at two additional mitochondrial genes for confirmation of species identification.

For genus-based identification of larvae, a forward primer, Mff18SF1 5' GGA TAA CTG TGG CAA TTC TAG 3' was designed by aligning the sequences of the near complete 18S rDNA of *Brugia malayi *[GenBank: AF036588], *Wuchereria bancrofti *[GenBank: AF227234], *D. immitis *[GenBank: AF036638] and *Dipetalonema *sp. [GenBank: DQ531723.1] using ClustalW (http://www.genome.jp/tools/clustalw/) and combined with reverse primer, PAFilariaR1 [20], to amplify a 700bp PCR product.

The PCR assay was carried out in a volume of 25 μl containing 1 × PCR buffer (Qiagen), 2.0 mM MgCl_2_, 200 μM of each dNTP, 0.5 μM of each of the forward and reverse primers, 0.5U of Taq DNA polymerase (Qiagen) and 1 μl of extracted DNA (concentration for each DNA samples ranged from 1.5 to 1.7 ng/μL). The PCR conditions were as follows: an initial activation step at 94°C for 2 min was followed by 35 cycles of amplification (94°C for 30s, 60°C for 30s and 72°C for 30s) followed by a final extension step of 72°C for 7 minutes.

The larvae were subjected to PCR to amplify the internal transcribed spacer-2 (ITS-2) region using PCR assays and conditions previously described by Rishniw [21]. Larvae and microfilariae were also subjected to PCR amplification of the mitochondrial 12S rDNA and cytochrome oxidase - 1 (*cox*-1) genes. Amplifications and sequences of 12S rDNA were generated using a primer pair 12SF and 12SR [23]. PCR was performed in a 25 μl final volume under the following conditions: 1× buffer A with 1.5 mM MgCl_2 _(KAPA2G™), 0.2 mM of each dNTP, 1 μM of each primer, and 0.75 U of KAPA2G™ Robust HotStart DNA Polymerase (KAPA2G™). The thermal profile used was: 95°C 30 s followed by 95°C 30 s, 50°C 15 s, and 72°C 30 s for 35 cycles and 72°C for 60 s. The *cox*-1 sequences were generated using the primer pair COIintF and COIintR using conditions described by Casiraghi and colleagues [24].

PCR products were purified using Qiagen spin columns (Qiagen) and sequenced using an ABI 3130xl Genetic Analyser (Applied Biosystems) with Big Dye 3.0 chemistry. The sequences were read, edited and assembled using Finch TV (Geospiza Inc.) and BioEdit Sequence Alignment Editor version 7.0.5.3.

Neighbor joining analyses were conducted with Tamura-Nei parameter distance estimates and trees constructed using Mega 4.1 software. Bootstrap analyses were conducted using 1000 replicates.

## Results

**Figure 2:**
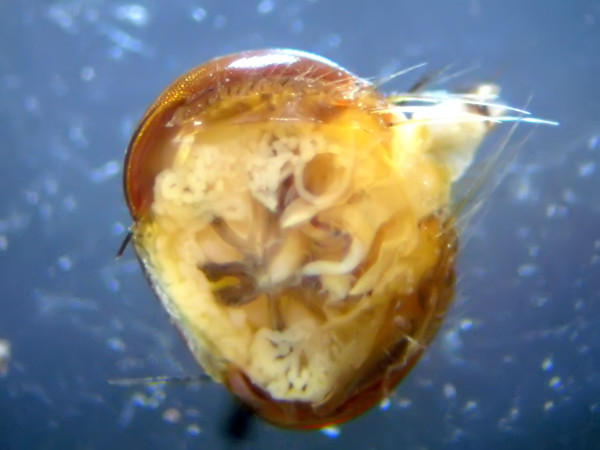
**An infective larva of *Acanthocheilonema *sp.? nov. in situ within the head of a *Hippobosca longipennis *fly**.

**Figure 3:**
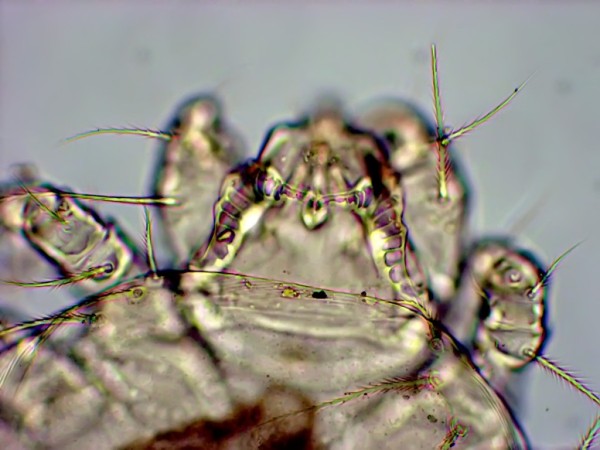
**The heart-shaped of the dorsal solenidion on genu I of the first leg confirms the mite species as *Cheyletiella yasguri***.

More than 200 flies were collected from a total of 101 dogs. From these a total of 65 flies were selected randomly and identified morphologically as *H. longipennis*. Twelve nematode larvae were found in 10 out of 65 flies dissected; 9 from the head (Figure 2), 2 from the thorax and 1 from the abdomen. Two flies were found with mites attached to the setae of their legs and these mites were identified as *Cheyletiella yasguri *(Figure 3) according to Domrow [25].

**Figure 4:**
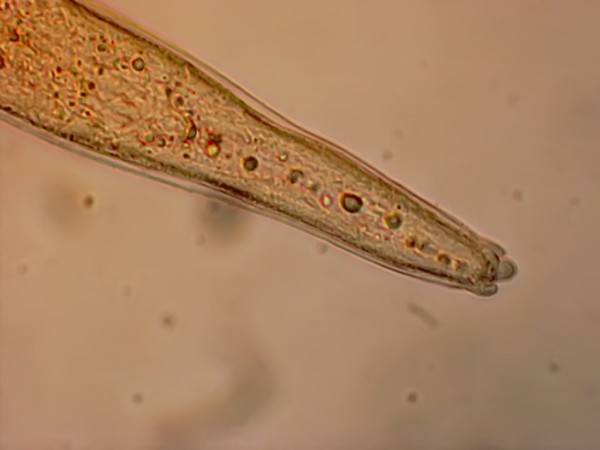
**The subterminal lateral caudal appendage of the infective larvae of *Acanthocheilonema *sp.? nov., a characteristic feature of the infective larvae from family *Onchocercidae***.

The larvae were relatively large, with a mean total body length of 2,900 (± 60) μm and width of 34 (± 5) μm. Apart from being larger, all extracted larvae closely resembled other infective larvae of the family Onchocercidae (species of the genera *Dirofilaria, Brugia, Cerchopithifilaria *and *Acanthocheilonema*), with a characteristic subterminal lateral caudal appendage (Figure 4), regardless of the location within the flies.

**Figure 5:**
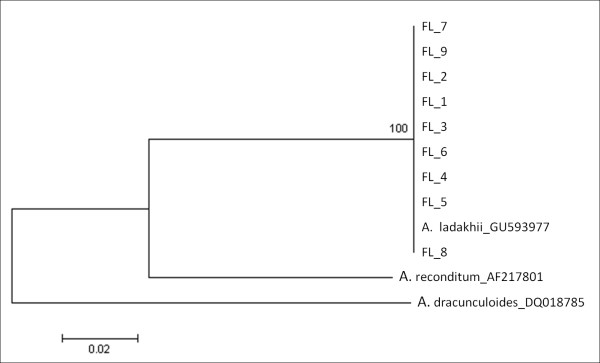
**Un-rooted phenogram construction of the ITS-2 gene showed 100% bootstrap placement for all nine *Acanthocheilonema *larvae isolated from *H. longipennis *sequences together with microfilaria sequence from previous study, using the Neighbour-Joining algorithm**. Bootstrap values at nodes indicate percentage calculated in 1000 replicates.

**Figure 6:**
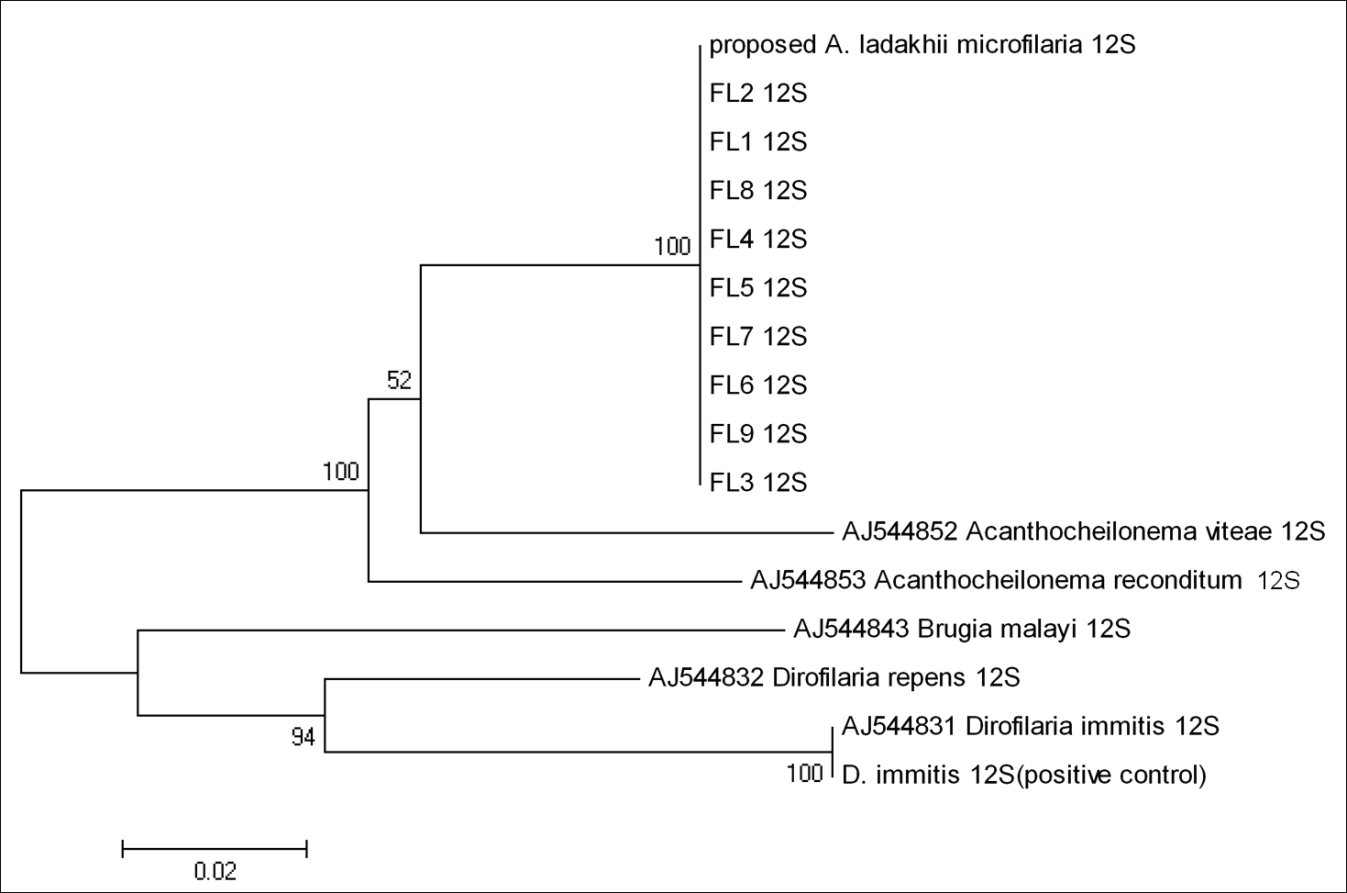
**Un-rooted phenogram construction of the 12S gene using the Neighbour-Joining algorithm**. Bootstrap values at nodes indicate percentage calculated in 1000 replicates. The phenogram showed 100% bootstrap placement for all nine *Acanthocheilonema *larvae isolated from *H. longipennis *sequences together with microfilaria sequence from previous study and forming a sister group with other species of *Acanthocheilonema*,

**Figure 7:**
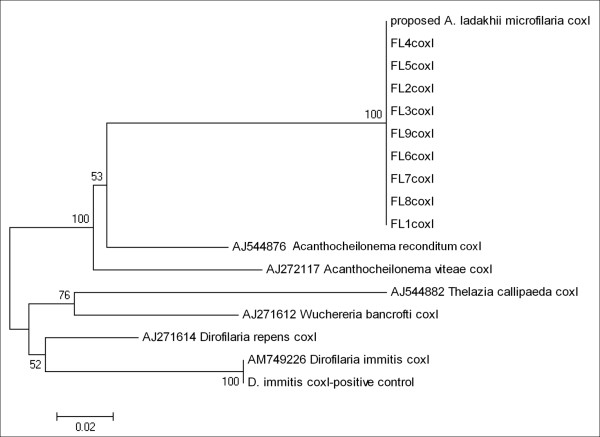
**Un-rooted phenogram construction of the cox-1 gene showed 100% bootstrap placement for all nine *Acanthocheilonema *larvae isolated from *H. longipennis *sequences together with microfilaria sequence from previous study and forming a sister group with other species of *Acanthocheilonema*, using the Neighbour-Joining algorithm**. Bootstrap values at nodes indicate percentage calculated in 1000 replicates.

Nine of the 11 larvae were subjected to PCR amplification at the 18S, ITS-2 genes, 12S and *cox*-1 genes. DNA sequences of all larvae displayed 100% homology to the *Acanthocheilonema *sp.? nov. at all four genetic loci. The un-rooted phenogram using neighbour joining analyses of the ITS-2, 12S and *cox*-1 sequences showed 100% bootstrap placement for all nine larvae sequences with *Acanthocheilonema *sp.? nov. into a distinct group from *A. reconditum *as well as *A. vitae *(Figure 5, 6, 7).

## Discussion

This study represents the first report of the dog louse fly, *H. longipennis*, acting as a potential intermediate host of the recently identified species of canine filarial nematode *Acanthocheilonema *sp.? nov.. Both conventional parasitological and molecular methods provide evidence of biological involvement between *H. longipennis *and this parasite in northern India. Despite careful examination of different anatomical regions of the flies during dissection, only infective larvae were observed; this is similar to the findings of Nelson [17] who also failed to find other developmental stages of larvae of *A. dracunculoides *except for a single second stage larva found in the abdominal cavity. Nelson suggested that the absence of other developmental stages may have been due to the prolonged feeding interval of the fly, which could allow larvae to become fully infective before re-feeding on the host a few days later. Hafez and Hilali [26] however demonstrated that the fly could only withstand starvation for 12 to 36 hours before dying and that feeding occurs more regularly than this. It is reported that the developmental period from L1 to infective larvae for *A. reconditum *in fleas and *A. dracunculoides *in ticks is approximately seven days and thirteen days, respectively [16,27]. The development of *Acanthocheilonema *sp.? nov. within its hippoboscid host therefore requires further investigation. Despite the fact that we have demonstrated a potential intermediate host-parasite relationship between *H. longipennis *and *Acanthocheilonema *sp.? nov., determination of the vector competence for any blood-feeding arthropod should be based on the demonstration of its capability of transmitting the parasite to a receptive host during blood feeding using experimental trials [15,27], and this remains to be performed.

The precise identification of this nematode remains unclear. A proper morphological comparison of the third-stage larvae, microfilaria and recovery and comparison of adult worms with those of the other filarial nematodes of dogs in addition to the molecular phylogenetics would be required in order to comply with the establishment criteria listed in Article 7 to 11 of the Code of Zoological Nomenclature, which would allow official classification of this *Acanthocheiloma *sp. nov.? found in dogs.

Species of *Hippobosca *do not normally travel long distances but they are strong fliers and will actively fly between hosts within a group [28]. When on the host, *H. longipennis *moves swiftly between hairs and is very difficult to catch (authors' personal observation). It is known that on average the female leaves the host for larviposition (on the soil, in crevices or in cracks of tree barks) about 8 times throughout her lifetime, on each occasion she returns to the host to feed and start another larval maturation cycle [26]. Hafez and Hilali [26] stated that the amount of complete blood meal ingested by *H. longipennis *varies from 1.5 to 4.5 mg with a feeding duration of 3 to 13 minutes and meals are taken at least every 6 hours. This information highlights the significance of this fly as important blood sucking ectoparasite, hence increasing the opportunity for it to be an effective disseminator of blood parasites among dogs.

The subfamilies Lipopteninae, and Ornithomyiinae are known biological vectors for *Trypanosoma *and *Haemoproteus *of birds, sheep and goat [2]. The subfamily Hippoboscinae has been documented only as a vector for *A. dracunculoides *in dogs [17]. More recently, over 70% of *Hippobosca equina *were positive for *Bartonella *DNA [29], but further investigation is needed to clarify biological or mechanical involvement of the fly as a vector for this pathogen. To the authors' knowledge, there are no other reports exploring the role of the genus *Hippobosca *as a vector/intermediate host for any other pathogen. Indeed, it is surprising how little information is known about the capability of the subfamily Hippoboscinae as vectors of pathogens. Further investigations are crucial to reduce this knowledge gap.

Despite being commonly encountered in semi-arid regions of Africa and Asia, there is no peer-reviewed information available on the efficacy of ectoparasitic drugs against *H. longipennis *on dogs. Staff at several zoos in United States applied methoxychlor, malathion and carbaryl-sulfur dust formulations to the animals and their surroundings to control and eradicate *H. longipennis *infestations [7,8]. In addition to its role as an intermediate host for canine filariae, our findings have also confirmed earlier reports that *C. yasguri *mites are phoretic on *H. longipennis *[30,31] and that the fly may play an important role in mechanically disseminating this mite among dogs. The fly's potential to cause heavy infections of dogs with accompanying anaemia and/or skin lesions, signals a need for further studies to determine the efficacy of currently available insecticides against *H. longipennis*.

With respect to the public health significance of this epifaunistic ectoparasite, it is known that humans are occasionally bitten by flies from the family Hippoboscidae [3], but it is of note that the authors did not receive any bites from *H. longipennis *during the collection process. Apart from mechanical irritation and injury, the fly may also facilitate the zoonotic transmission of *C. yasguri *[32]. It has also been hypothesised that *H. longipennis *may act as a mechanical vector for *Leishmania *spp. in areas where the parasite is endemic in dogs [8]. Furthermore, *H. equina *(forest fly) and *Lipoptena cervi *(deer ked), other blood-sucking species belonging to the family Hippoboscidae, have been reported feeding on [33] and causing anaphylactic reactions in humans in Finland [34] and Hungary [35] and this may also be a possibility with *H. longipennis*. In summary, to date, this fly appears to have minimal public health significance, but further investigation is needed to verify this.

## Conclusion

This study confirmed biological involvement of *H. longipennis *for *Acanthocheilonema *sp.? nov. in northern India and its role as an intermediate host is proposed. Further investigations are essential to prove whether this fly is a competent vector for *Acanthocheilonema *sp.? nov.. Furthermore, future research needs to be conducted to investigate whether *H. longipennis *is capable of propagating and transmitting other vector-borne and zoonotic pathogens.

## Competing interests

The authors declare that they have no competing interests.

## Authors' contributions

PAMAR was involved in all phases of the study, including sampling and data collection, laboratory work, data analysis, intellectual interpretation, and writing the manuscript.

RJT designed the study project, supervised the study, and was involved in sampling, field data collection, intellectual interpretation and critical revision of the manuscript for publication.

PJI and GTC supervised the study and were involved in intellectual interpretation and critical revision of the manuscript for publication. All authors read and approved the final manuscript.
